# LTRs of Endogenous Retroviruses as a Source of Tbx6 Binding Sites

**DOI:** 10.3389/fchem.2017.00034

**Published:** 2017-06-15

**Authors:** Yukuto Yasuhiko, Yoko Hirabayashi, Ryuichi Ono

**Affiliations:** Division of Cellular and Molecular Toxicology, Biological Safety Research Centre, National Institute of Health SciencesTokyo, Japan

**Keywords:** endogenous retroviruses, retrotransposon, transcription factors, evolution, TBX6

## Abstract

Retrotransposons are abundant in mammalian genomes and can modulate the gene expression of surrounding genes by disrupting endogenous binding sites for transcription factors (TFs) or providing novel TFs binding sites within retrotransposon sequences. Here, we show that a (C/T)CACACCT sequence motif in ORR1A, ORR1B, ORR1C, and ORR1D, Long Terminal Repeats (LTRs) of MaLR endogenous retrovirus (ERV), is the direct target of Tbx6, an evolutionary conserved family of T-box TFs. Moreover, by comparing gene expression between control mice (Tbx6 +/−) and Tbx6-deficient mice (Tbx6 −/−), we demonstrate that at least four genes, *Twist2, Pitx2, Oscp1*, and *Nfxl1*, are down-regulated with Tbx6 deficiency. These results suggest that ORR1A, ORR1B, ORR1C and ORR1D may contribute to the evolution of mammalian embryogenesis.

## Introduction

About half of the mammalian genome is occupied by DNA sequences derived from transposable elements (TEs) (Lander et al., 2001; Waterston et al., 2002; Lindblad-Toh et al., 2005; de Koning et al., 2011). Retrotransposons, which mobilize via an RNA intermediate by a copy-and-paste mechanism, comprise the majority of mammalian TEs, whereas DNA transposons, which move via a cut-and-paste mechanism, comprise a small fraction and have accumulated mutations that render them immobile (Deininger et al., 2003). Most TEs are nonfunctional and are regarded as genomic parasites or junk DNA; however, a growing body of evidence suggests that retrotransposons and retrotransposon-derived genes have acquired functions essential for host survival during mammalian evolution (Yoder et al., 1997; Levin and Moran, 2011; Hancks and Kazazian, 2012).

In some cases, open reading frames from TEs are domesticated as endogenous genes during mammalian evolution. For example, *Peg10* and *Rtl1*, derived from the gag and pol proteins of the Ty3/Gypsy type retrotransposon, which is similar to *Sushi-ichi*, are highly conserved in mammals and participate in placental formation (Ono et al., 2001, 2003, 2006; Sekita et al., 2008). Similar to the gag protein of *Sushi-ichi*, the other two of the eleven *Sushi-ichi retrotransposon homolog* (*Sirh*) family genes, *Sirh7/Ldoc1* and *Sirh11/Zcchc16*, encode ORF (Open-Reading frame); they are also involved in the determination of the timing of parturition and cognitive function in the brain, respectively (Ono et al., 2011; Naruse et al., 2014; Irie et al., 2015). *Syncytins/SYNCYTINs* (mouse/human) and *FEMATRIN* (cow), derived from the envelope of endogenous retrovirus (ERV), mediate cell-cell fusion to form the syncytiotrophoblast and induce fusion with bovine endometrial cells *in vitro* (Mi et al., 2000; Dupressoir et al., 2009, 2011; Nakaya et al., 2013). *Skin aspatic protease* (*SAPase*), which has a retrovirus-like aspartic protease, plays important roles in the determination of the texture of the skin by modulating the degree of hydration by processing profilaggrin (Matsui et al., 2010).

Since the discovery of TEs, it has been posited that TEs may seed regulatory elements throughout genomes and drive phenotypic differences between species via changes in transcriptional output (McClintock, 1950; Britten and Davidson, 1969; Feschotte, 2008). It has become evident that many TEs, such as long terminal repeats (LTRs) of endogenous retroviruses (ERVs), contain TF binding sites and are associated with gene expression patterns. For example, MuERV-L LTRs function as alternative promoters for protein coding genes, including *Gata4* and *Tead4*, which are important for the specification of primitive endoderm and trophectoderm, respectively, in two-cell embryos (Kigami et al., 2003; Evsikov et al., 2004; Macfarlan et al., 2012). It has also been reported that MuERV-L, exclusively expressed in two-cell embryos, is captured at double-strand break (DSB) sites introduced by the CRISPR/Cas9 system in mouse zygotes (Ono et al., 2015). Some of the intracisterminal A-particle (IAP) retrotransposon insertions are known to induce *de novo* metastable epi-alleles, such as *agouti viable yellow* (*Avy*), *axin fused* (*AxinFu*) and *Cdk5rap* locus (Vasicek et al., 1997; Morgan et al., 1999; Druker et al., 2004). The stochastic nature of the establishment of the epigenetic state of the 5′ LTR leads to variable expressivity of the adjacent genes. Both the sense and anti-sense LINE-1 (L1) promoter can drive L1 chimeric transcripts (Criscione et al., 2016). Moreover, AS071 and AS021, two AmnSINE1s present in mammals as well as birds and reptiles, are enhancers of the genes *FGF8* (fibroblast growth factor 8), 178 kb from AS071, and *SATB2*, 392 kb from AS021 (Sasaki et al., 2008). Recently, it was reported that MER41, a primate-specific endogenized gammaretrovirus, is a source of interferon γ (IFNG)-inducible binding sites (Chuong et al., 2016).

In this study, we demonstrate a potential role for ORR1A (Origin-Region Repeat 1A), ORR1B, ORR1C, and ORR1D, LTRs of the MaLR (Mammalian-Apparent Long-Terminal Repeat Retrotransposon) endogenous retrovirus-like element, in controlling gene expression via *Tbx6* binding (Smit, 1993). Because *Tbx6* functions in the regulation of early embryogenesis, including anti-neural fate regulation in the presomitic mesoderm and later somite segmentation, ORR1A, ORR1B, ORR1C, and ORR1D may have played a role in the evolution of mammalian embryogenesis (Chapman and Papaioannou, 1998; Takemoto et al., 2011).

## Results and discussion

*Tbx6* belongs to an evolutionarily conserved family of T-box transcription factors (TFs), known to be involved in the neural-mesodermal fate determination of axial stem cells (Chapman and Papaioannou, 1998; Takemoto et al., 2011). Previously, we revealed that Tbx6 directly activates the expression of *Mesp2*, a segmentation and polarization factor in somitogenesis, in a Notch signal-dependent manner (Yasuhiko et al., 2006). A ligand of Notch signal, Dll1, is also a direct target of Tbx6, implying that Tbx6 participates in the regulation of the Notch signaling pathway (White and Chapman, 2005). The consensus core sequence of Tbx6 binding sites has been reported as CACACCT or AGGTGTBRNNNN (White and Chapman, 2005). In this study, we used (C/T)CACACCT as a consensus for both reports (White and Chapman, 2005; Yasuhiko et al., 2006).

**Figure 1:**
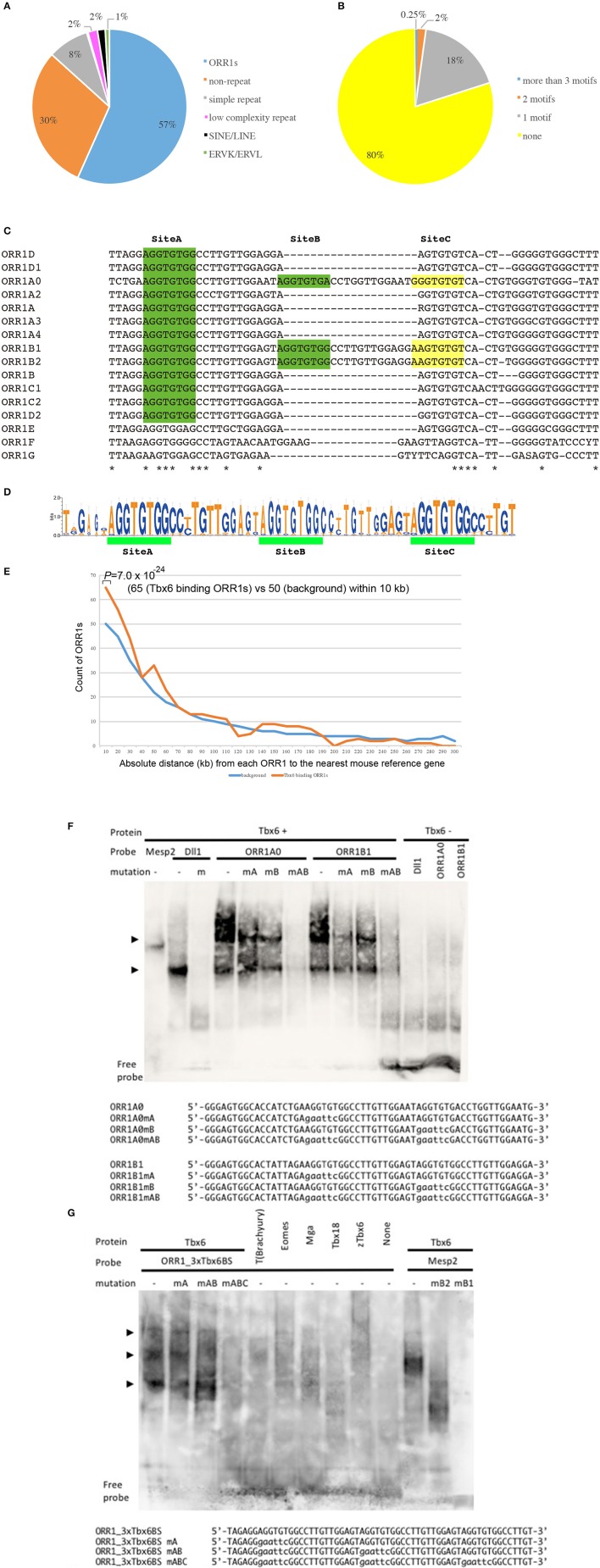
Tbx6 binding sequence motifs in LTRs of ORR1 ERV families. **(A)** Distribution of Tbx6 binding sequence motifs in the mouse genome (MM10). Of the 3,500 Tbx6 binding sequence motifs, 70% were repeat sequences, including TEs and simple repeats. ORR1, Long Terminal Repeats (LTRs) of MaLR endogenous retrovirus (ERV), occupy 57% of the total Tbx6 binding sequence motifs. **(B)** Of all the ORR1 sequences in the mouse genome, 20% of ORR1s have at least one Tbx6 binding sequence. **(C)** DNA sequence comparison between ORR1A (rodentia ancestral shared), ORR1A0 (mus musculus), ORR1A2 (muridae), ORR1A3 (muridae), ORR1A4 (muridae), ORR1B (rodentia ancestral shared), ORR1B1 (mus musculus), ORR1B2 (mus musculus), ORR1C1 (rodentia ancestral shared), ORR1C2 (rodentia ancestral shared), ORR1D1 (rodentia ancestral shared), ORR1D2 (rodentia ancestral shared), ORR1E (rodentia ancestral shared), ORR1F (muridae) and ORR1G (muridae) LTRs. Identical sequences are indicated by asterisks. The Tbx6 binding sequence motif is indicated by green boxes. Yellow boxes are indicated as a corresponding region of the Tbx6 binding sequence motif “Site C” in Figure 1D. **(D)** Sequence logo of the the ORR1 LTRs that had more than three Tbx6 binding sequence motifs. Three tandem “AGGTGTGs,” a Tbx6 binding sequence motif, are highly conserved between ORR1 LTRs, which have more than three Tbx6 binding sequence motifs. **(E)** Frequency histogram of the absolute distance from each ORR1 to the nearest mouse reference gene. The background expectation is derived from the genome-wide ORR1s distribution. Statistical significance of the observed enrichment within the first 10 kb of the nearest mouse reference gene was assessed by a binominal test. **(F)** Site A and site B sequences independently bind to Tbx6 in an electromobility shift assay (EMSA); however, the binding affinity is much higher with the presence of both sites A and B. Sequences of oligonucleotide probes were shown below the gel image. Mutated nucleotides were depicted in lower case. **(G)** Triple Tbx6 binding sequence motif shows the highest binding affinity to Tbx6, while other T-box TFs, including T (Brachyury), Eomes, Mga, Tbx18, and zebrafish Tbx6 (zTbx6), have no affinity. Arrowheads in **(F,G)**: Positions of the bands resulted from multiple Tbx6 binding to ORR1 sequences. Sequences of oligonucleotide probes were shown below the gel image. Mutated nucleotides were depicted in lower case.

At first, the Tbx6 binding sequence motif, (C/T) CACACCT, was identified by whole genome *in silico* screening. Furthermore, we chose the *Tbx6* binding sequence, which has at least two more Tbx6 binding sequences within the neighboring 100 bp upstream and/or downstream regions, because we previously demonstrated that higher enhancer activity of Tbx6 was observed when there are more than three Tbx6 binding sequences within a narrow region. As a result, 3500 potential Tbx6 binding sites were identified, and a characteristic feature was revealed (Figure 1A; Supplementary Table 1).

Approximately 70.0% of potential Tbx6 binding sites comprise repeat sequences (Figure 1A). Specifically, 85.7% of the potential Tbx6-binding-repeat sequences were within ORR1A, ORR1B, ORR1C, and ORR1D, LTRs of the MaLR that span 679 independent ORR1s-LTRs, while SINEs and LINES represent only 2% of the Tbx6-binding repeat sequences (Bao et al., 2015; Supplementary Table 1).

There are 166,375 Repeatmasker annotated ORR1s, including partial sequences, in the mouse genome (MM10), and 20% of them have at least one Tbx6 binding site (Figure 1B). In fact, the reference sequences of ORR1s-LTRs from Repbase, which are consensus sequences of ORR1s, have one or two Tbx6 binding sequence motifs (Figure 1C). These data suggest that the tandem insertion of these LTRs or degenerated LTR sequences with more than three Tbx6 binding sequence motifs might be good targets for *Tbx6* to bind *in vivo*. Furthermore, potential Tbx6-binding ORR1s have more than three Tbx6 binding motifs within themselves or share the Tbx6 binding motifs with neighboring sequences.

Tbx6-binding ORR1s more than 300 bp in length were selected, and the consensus sequences including three Tbx6-binding motifs and the absolute distance from each ORR1 to the nearest mouse reference gene were determined (Figures 1D,E; Supplementary Table 1). The strong interaction between Tbx6 and the consensus sequence of Tbx6-binding ORR1s were confirmed by electrophoretic mobility shift assay (EMSA), while the interactions disappeared by introducing mutations into the Tbx6-binding motif one by one (Figure 1F). The finding that three Tbx6 binding motifs rather than one or two Tbx6 binding motifs have stronger binding affinity was comparable to our previous report (Figure 1G; Yasuhiko et al., 2008). As Tbx6-binding ORR1s were relatively enriched near gene transcription start sites (Figure 1E), Tbx6 may contribute to regulating the expression level of nearby genes until reaching 60 kb-windows. Then, to explore the influence of ORR1A, ORR1B, ORR1C, and ORR1D on the regulation of gene expression by *Tbx6*, we compared the expression level of 9 genes that are randomly selected within 50 kb of potential Tbx6 binding sites on ORR1A, ORR1B, ORR1C, and/or ORR1D in *Tbx6* (+/−) (control) and *Tbx6* (−/−) (*Tbx6* KO) embryos at 8.0 day post-coitus (dpc). Because *Tbx6* KO embryos have morphological abnormalities after 9 dpc, we used 8.0 dpc embryos in this study to exclude secondary effects from morphological abnormalities.

**Figure 2:**
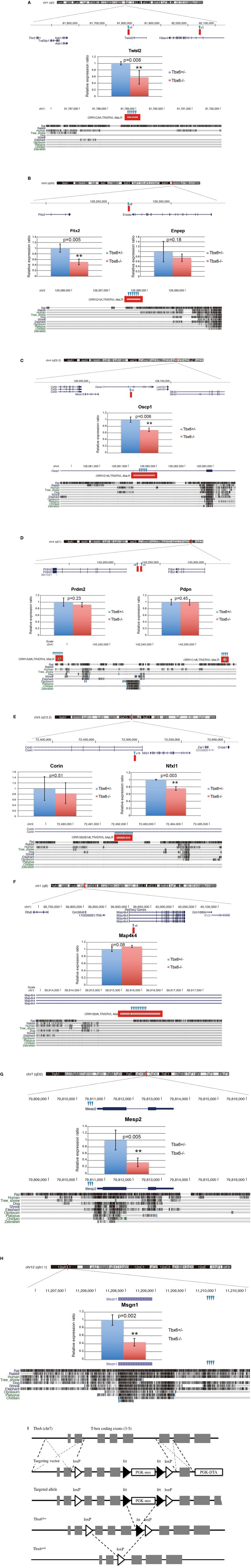
ORR1 family of ERVs function as enhancers by Tbx6-binding. Positions of LTRs including potential Tbx6 binding sites (red bars) are indicated in the upper side of each panel **(A–F)**. Relative expression levels were determined by quantitative RT-PCR of the Tbx6-binding-LTRs adjacent genes *Twist2* **(A)**, *Pitx2* **(B)**, *Enpep* **(B)**, *Oscp1* **(C)**, *Prdm2* **(D)**, *Pdpn* **(D)**, *Nfxl1* **(E)**, and *Map4k4* **(F)** and the previously reported positive control genes *Mesp2* **(G)** and *Msgn1* **(H)** in *Tbx6* (+/−) (control: blue bars) and *Tbx6* (−/−) (Tbx6 KO: red bars) embryos at 8.0 dpc. The relative expression ratios are normalized to the housekeeping gene β*-actin*. The details of Tbx6-binding LTRs and conservation among vertebrate species. The Tbx6-binding LTRs (red boxes) are also conserved in rats (bottom of **A–C**); however, another three are not conserved in other rodents (bottom of **D–F**). Tbx6-binding motifs are indicated by blue triangles. Schematic description of *Tbx6* conditional KO mouse was shown in **(I)**.

As expected, four genes, *Twist2, Pitx2, Oscp1*, and *Nfxl1*, were down-regulated, although the expression of five other genes, *Enpep, Prdm2, Corin, Pdpn* and *Map4k4*, was not altered significantly (Figure 2). It has been reported that enhancer activity could be blocked by the epigenetic repressive marks of the neighboring regions, such as histone deacetylation and tri-methylation of K9 and K27 on histone H3 (H3K27me3 and H3K9me3) or an insulator, a genetic boundary element blocking the interaction between enhancers and promoters (Roth et al., 2001; Schmidl et al., 2009; Greer and Shi, 2012; Dowen et al., 2014). It might be possible that five genes whose expression levels were not altered by Tbx6 deficiency could be blocked by epigenetic modifications or unknown silencers.

**Figure 3:**
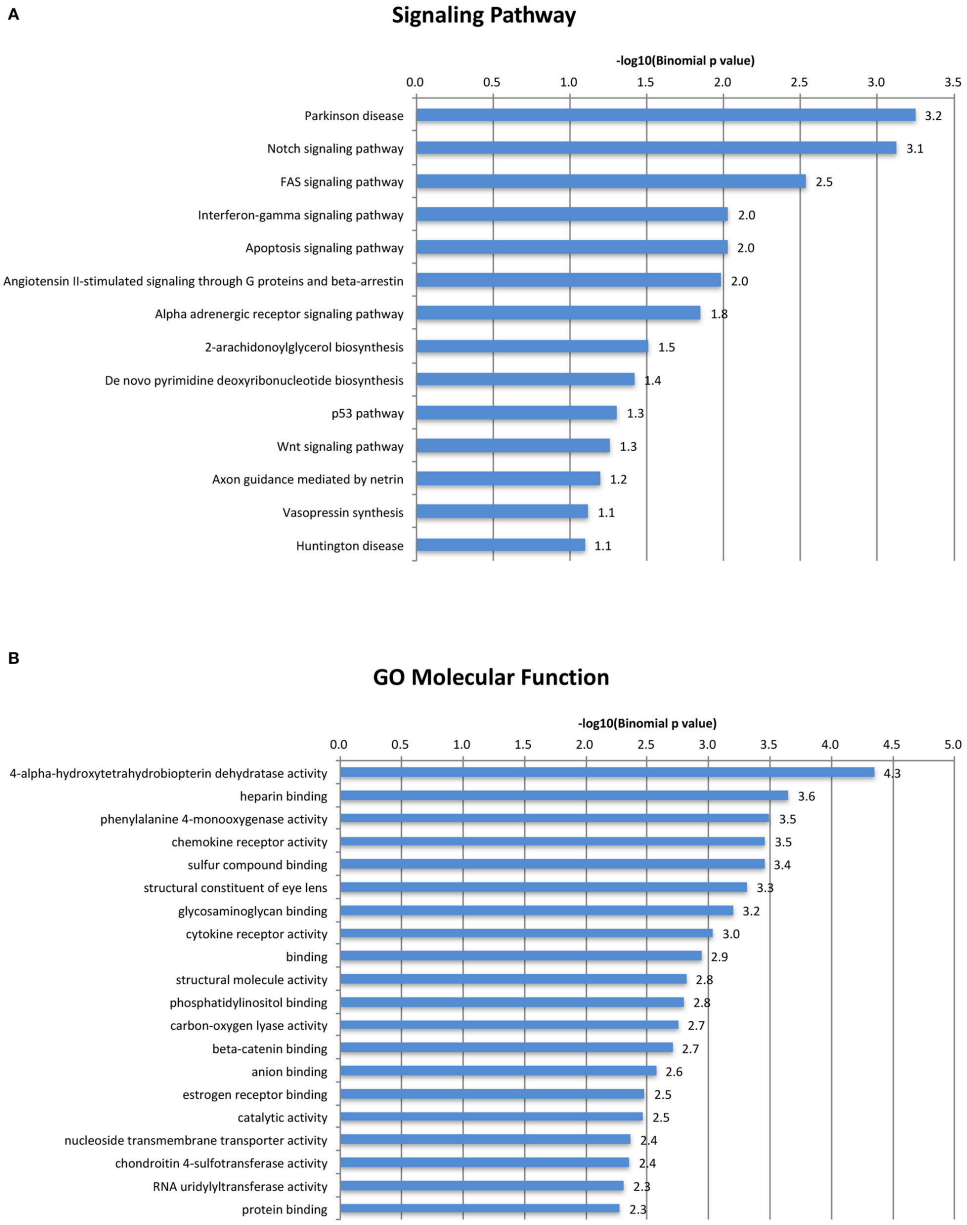
A gene ontology/signal pathway panel of Tbx6-binding ORR1s. Top categories of GO Biological Process were shown. **(A)** Top categories of Signal Pathway were shown. **(B)** All displayed categories were significant by binomial test (*P* < 0.1).

Our analysis revealed the rodent-specific ORR1 family of ERVs to be a source of Tbx6 binding sites. Furthermore, Tbx6-binding ORR1s are enriched near genes which might be associated with several biological process and molecular pathways (Figure 3). In the human genome, there are 2,927 potential TBX6 binding motifs; however, the majority of sites are not in LTRs but in simple repeat sequences or Alu (Supplementary Table 2). Although the source of Tbx6/TBX6 binding sequences is different between species, each mammalian species might shape their Tbx6/TBX6 binding sequence through mammalian evolution. Our analysis and other reports, including the primate-specific MER41 family as IFNG-inducible binding sites and AmnSINE1s as mammalian enhancers, raised the possibility that TE-derived regulatory elements influence lineage-specific mammalian evolution (Sasaki et al., 2008; Chuong et al., 2016).

## Materials and methods

### Bioinformatic analyses

(C/T)CACACCT, Tbx6 binding sequence motifs, were identified in the mouse whole genome (MM10) and human whole genome (hg19) and filtered out when there were not two more Tbx6 binding sequences within the neighboring 100 bp upstream and/or downstream regions using gggenome (https://gggenome.dbcls.jp). All the TE sequences were downloaded from Repeatmasker truck (mouse:MM10/human:hg19) of the UCSC genome browser (https://genome.ucsc.edu). The Intersect intervals program (https://usegalaxy.org/) was used to identify the TEs that have potential Tbx6 binding sequences using potential Tbx6 binding sites identified as a query against Repeatmasker annotated TEs. The ClosestBed program (https://usegalaxy.org/) was used to find the closest mouse reference genes (MM10) and to identify the absolute distance between the potential Tbx6 binding motif and its closest reference gene. These distances were grouped by 10 kb-bin sizes. The expected background was determined by randomly sampling an equal number of the remaining 78,042 annotated ORR1s that did not have more than three Tbx binding motifs. Sampling was repeated 100 times, and the mean number of elements was used as the expected value for comparison to the potential Tbx6 binding ORR1s. Statistical significance was determined for the first 10-kb bin by a binominal test as previously described (Chuong et al., 2016). Gene ontology of the closest reference genes within 50 kb-windows of potential Tbx6-binding motifs were determined by the GREAT program http://bejerano.stanford.edu/great/public/html/index.php (Figure 3). The consensus sequence of the potential Tbx6-binding motifs was identified by ClustalW program (for alignment: http://clustalw.ddbj.nig.ac.jp/index.php?lang=ja)and Sequence Logo program (for generation of sequence logos: http://weblogo.berkeley.edu). A gene ontology/signal pathway panel of Tbx6-binding ORR1s.

### Electrophoretic mobility shift assay (EMSA)

Full sequences of ORFs of mouse *Tbx6* (NM_011538.2), *T* (*Brachyury*; NM_009309.2), *Eomes* (NM_010136.3), *Tbx18* (NM_023814.4), and T-box-coding fragment of Mga (NM_013720.2) were PCR amplified and cloned in pCS2+ (Rupp et al., 1994) vector. Expression vector pCS2-zTbx6 for zebrafish Tbx6 translation was a gift from Dr Hiroyuki Takeda (Terasaki et al., 2006). Transcription factors were *in vitro* transcribed and translated using TnT(R) Quick Coupled Transcription/Translation System (Promega) following the manufacturer's protocol. Sequences of DNA probes were as follows: Mutated nucleotides are designated in lower case. Mesp2 and Dll1 were positive controls for the assay and described in Yasuhiko et al. (2006) and White and Chapman (2005), respectively. 1ORR1A0, 5′-GGGAGTGGCACCATCTGAAGGTGTGGCCTTGTTGGAATAGGTGTGACCTGGTTGGAATG-3′; ORR1A0mA, 5′-GGGAGTGGCACCATCTGAgaattcGGCCTTGTTGGAATAGGTGTGACCTGGTTGGAATG-3′; ORR1A0mB, 5′-GGGAGTGGCACCATCTGAAGGTGTGGCCTTGTTGGAATgaattcGACCTGGTTGGAATG-3′; ORR1A0mAB, 5′-GGGAGTGGCACCATCTGAgaattcGGCCTTGTTGGAATgaattcGACCTGGTTGGAATG-3′; ORR1B1, 5′-GGGAGTGGCACTATTAGAAGGTGTGGCCTTGTTGGAGTAGGTGTGGCCTTGTTGGAGGA-3′; ORR1B1mA, 5′-GGGAGTGGCACTATTAGAgaattcGGCCTTGTTGGAGTAGGTGTGGCCTTGTTGGAGGA-3′; ORR1B1mB, 5′-GGGAGTGGCACTATTAGAAGGTGTGGCCTTGTTGGAGTgaattcGGCCTTGTTGGAGGA-3′; ORR1B1mAB, 5′-GGGAGTGGCACTATTAGAgaattcGGCCTTGTTGGAGTgaattcGGCCTTGTTGGAGGA-3′; ORR1_3xTbx6BS, 5′-TAGAGGAGGTGTGGCCTTGTTGGAGTAGGTGTGGCCTTGTTGGAGTAGGTGTGGCCTTGT-3′; ORR1_3xTbx6BSmA, 5′-TAGAGGgaattcGGCCTTGTTGGAGTAGGTGTGGCCTTGTTGGAGTAGGTGTGGCCTTGT-3′; ORR1_3xTbx6BSmAB, 5′-TAGAGGgaattcGGCCTTGTTGGAGTgaattcGGCCTTGTTGGAGTAGGTGTGGCCTTGT-3′; ORR1_3xTbx6BSmABC, 5′-TAGAGGgaattcGGCCTTGTTGGAGTgaattcGGCCTTGTTGGAGTgaattcGGCCTTGT-3′; Mesp2, 5′-CCTTCGAGGGGTCAGAATCCACACCTCTGCAAATGGGCCCGCTTT-3′; Mesp2mB2, 5′- CCTTCGAGaGtaCtGAATCCACACCTCTGCAAATGGGCCCGCTTT-3′; Mesp2mB1, 5′- CCTTCGAGGGGTCAGAATCgAtAtCTCTGCAAATGGGCCCGCTTT-3′; Dll1, 5′-ACAATCAAAGGAACACTAGCTCCAAGAATCACACCTCGGGATTCTAATGAAGCTGCCTA-3′; Dll1m, 5′-ACAATCAAAGGAACACTAGCTCCAAGAATCgaattcCGGGATTCTAATGAAGCTGCCTA-3′. Sense and anti-sense oligonucleotides for each probe were annealed, DIG-labeled and subjected to EMSA assay using the DIG Gel Shift Kit, 2nd Generation (Roche). Briefly, 4 fmol of labeled oligonucleotide probe was incubated with 4 μl of *in vitro* translated mixture, electrophoresed in 7.5% polyacrylamide gel for 80 min at 100 V, and blotted to a positively charged nylon membrane. Shifted oligonucleotides were detected using an anti-DIG Fab fragment (Roche) and CDP-Star Ready-to-Use AP substrate (Roche).

### Gene targeting, mouse embryos and real-time RT-PCR

All animal studies were conducted in accordance with the guidelines approved by the animal care committee of the National Institute of Health Sciences (NIHS; No.934). The protocol was approved by the animal welfare committee of National Institute of Health Sciences (NIHS; No.41). Animals had access to a standard chow diet and water *ad libitum* and were housed in a pathogen-free barrier facility with a 12L:12D cycle.

A Tbx6 conditional knockout mouse (Tbx6flox) was generated using the ES cell line TT2 and maintained in an ICR background (Yagi et al., 1993). Briefly, exon 3-5, encoding the T-box DNA binding domain of Tbx6, was flanked by a pair of loxP sites and knocked into the *Tbx6* locus by homologous recombination. The PGK-neo selection marker was removed by the FLP-FRT system to obtain *Tbx6*flox mice. For cDNA preparation, embryos (8.0 days post-coitus) were obtained by crossing female CAG-*Cre*/*Tbx6*flox/+ hybrid heterozygotes onto male *Tbx*6flox/flox homozygotes. Embryos were genotyped by PCR using allantois genomic DNA, and total RNA were prepared using an RNeasy mini kit (QIAGEN). Total RNA was pooled from 5 (Tbx6+/−) and 4 (Tbx6−/−) 8.0dpc sibling embryos in the same litter. The sequences of primers for real-time RT-PCR were as follows:

*Twist2*_forward, 5′-TGTCCGCCTCCCACTAGC-3′;

*Twist2*_reverse, 5′-TGTCCAGGTGCCGAAAGTC-3′;

*Pitx2*_forward, 5′-GGCAGTCACCCTGGGAAG-3′;

*Pitx2*_reverse, 5′-GCCGACACTAGTTTGCGACA-3′;

*Enpep*_forward, 5′-CCTGCTTTACGACCCCCTAC-3′;

*Enpep*_reverse, 5′-TTAGCCACAAGTCGTCCCAC-3′;

*Oscp1*_forward, 5′-GACTCTGCCGCTGCTCT-3′;

*Oscp1*_reverse, 5′-TCGTCCATGAACTTCCTGTTGA-3′;

*Prdm2*_forward, 5′-GCTTCGAGGACTTCCAGAGG-3′;

*Prdm2*_reverse, 5′-TGGTTTAGTGGCCCAGACAC-3′;

*Pdpn*_forward, 5′-AGGTGCTACTGGAGGGCTTA-3′;

*Pdpn*_reverse, 5′-GCTGAGGTGGACAGTTCCTC-3′;

*Nfxl1*_forward, 5′-AGAACCTCCTCAGTTGCTGC-3′;

*Nfxl1*_reverse, 5′-AAGGGGCATTCACCAGGATG-3′;

*Corin*_forward, 5′-GATATGTTCACGAAACGGCCC-3′;

*Corin*_reverse, 5′-CGCTCCTGTCTGCTCTCAAG-3′;

*Map4k4*_forward, 5′-TTCCGGCCTCTCAAGCCT-3′;

*Map4k4*_reverse, 5′-TCCCAGACTCCTCACTGGAG-3′;

*Mesp2*_forward, 5′-ACCCTTACACCAGTCCCTAGAAA-3′;

*Mesp2*_reverse, 5′-GGTTCTGGAGACACAGAAAGACT-3′;

*Msgn1*_forward, 5′-GCCAGAAAGGCAGCAAAGTC-3′;

*Msgn1*_reverse, 5′-AGACAGGCGGCAGGTAATTC-3′;

β *-actin*_forward, 5′-CTGTCGAGTCGCGTCCA-3′;

β*-actin*_reverse, 5′-ACGATGGAGGGGAATACAGC-3′;

Primers were designed using Primer-BLAST (https://www.ncbi.nlm.nih.gov/tools/primer-blast/) tool to amplify 70–150 base pair (bp) fragment separated by at least one intron (>500 bp), except Msgn1 (single exon gene). PCR reaction was performed using SYBR(R) Premix Ex Taq(TM) II (Takara RR820S) following the manufacturer's protocol, with PCR cycle as follows: 1 cycle of 95°C 30 s, 40 cycles of 95°C 5 s and 60°C 30 s.

### Statistical analyses

Statistical significance for qPCR was assessed using a two-tailed unpaired Student's *t*-test with a threshold of *p* < 0.1.

## Ethics statement

The animal facility of the National Institute of Health Sciences was approved by the Japan Health Sciences Foundation since 2008. All animal studies were conducted in accordance with the guidelines approved by the animal welfare committee of the National Institute of Health Sciences (NIHS; No. 41).

## Author contributions

RO conceived of the project. YY, YH, and RO participated in the experimental design. RO performed most analyses. YY produced Tbx6 KO mice and performed EMSA and RT-PCR. RO wrote the manuscript. All authors read and approved the final manuscript.

### Conflict of interest statement

The authors declare that the research was conducted in the absence of any commercial or financial relationships that could be construed as a potential conflict of interest.
